# 2-(4-Acetamido­phen­oxy)-2-methyl­propanoic acid

**DOI:** 10.1107/S1600536813004856

**Published:** 2013-02-28

**Authors:** Gabriel Navarrete-Vázquez, Blanca Colín-Lozano, Hugo Tlahuext, Antonio R. Tapia-Benavides

**Affiliations:** a Facultad de Farmacia, Universidad Autónoma del Estado de Morelos, Av. Universidad 1001 Col., Chamilapa, CP 62100, Cuernavaca Mor., Mexico; b Centro de Investigaciones Químicas, Universidad Autónoma del Estado de Morelos, Av. Universidad 1001 Col., Chamilpa, CP 62100, Cuernavaca Mor., Mexico; c Centro de Investigaciones Químicas, Universidad A. del Estado de Hidalgo, Carr. Pachuca-Tulancingo Km. 4.5, Mineral de la reforma, CP 42184, Hidalgo, Mexico

## Abstract

In the title compound, C_12_H_15_NO_4_, the dihedral angle between the acetamide group and the ring is 29.6 (2)(su?)°. In the crystal mol­ecules are linked through N—H⋯O and O—H⋯O hydrogen bonds, thereby forming corrugated sheets propagating in the *ac* plane. These sheets are composed of *R*_4_^4^(28) graph-set motifs.

## Related literature

For related literature on analogous structures with analgesic and anti­dyslipidemic activities, see: Kis *et al.* (2005 ▶); Navarrete-Vázquez *et al.* (2008 ▶, 2011 ▶); Thorp & Waring (1962 ▶); Miller & Spence (1998 ▶); Forcheron *et al.* (2002 ▶). For information on hydrogen bonding, see: Bernstein *et al.* (1995 ▶); Jeffrey (1997 ▶); Desiraju (1996 ▶).
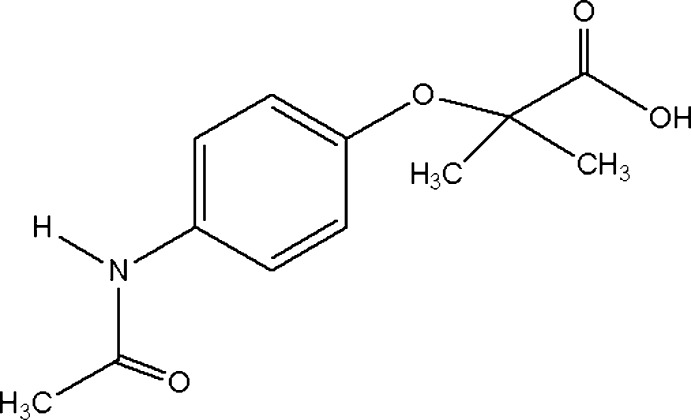


## Experimental

### 

#### Crystal data

C_12_H_15_NO_4_
*M* *_r_* = 237.25
Monoclinic, 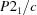

*a* = 8.3184 (4) Å
*b* = 13.1554 (6) Å
*c* = 12.0452 (5) Å
β = 109.959 (5)°
*V* = 1238.96 (10) Å^3^
*Z* = 4
Mo *K*α radiation
μ = 0.10 mm^−1^
*T* = 293 K
0.19 × 0.14 × 0.13 mm

#### Data collection

Agilent Xcalibur Atlas Gemini diffractometer
Absorption correction: multi-scan (*CrysAlis PRO*; Agilent, 2011 ▶) *T*_min_ = 0.982, *T*_max_ = 0.988
34747 measured reflections
2179 independent reflections
1738 reflections with *I* > 2σ(*I*)
*R*_int_ = 0.045

#### Refinement

*R*[*F* ^2^ > 2σ(*F* ^2^)] = 0.037
*wR*(*F* ^2^) = 0.098
*S* = 1.04
2179 reflections
161 parameters
1 restraint
H atoms treated by a mixture of independent and constrained refinement
Δρ_max_ = 0.20 e Å^−3^
Δρ_min_ = −0.18 e Å^−3^

### 

Data collection: *CrysAlis PRO* (Agilent, 2011 ▶); cell refinement: *CrysAlis PRO*; data reduction: *CrysAlis PRO*; program(s) used to solve structure: *SHELXS97* (Sheldrick, 2008 ▶); program(s) used to refine structure: *SHELXL97* (Sheldrick, 2008 ▶); molecular graphics: *SHELXTL* (Sheldrick, 2008 ▶); software used to prepare material for publication: *PLATON* (Spek, 2009 ▶) and *DIAMOND* (Crystal Impact, 2006 ▶).

## Supplementary Material

Crystal structure: contains datablock(s) I, global. DOI: 10.1107/S1600536813004856/gw2131sup1.cif

Structure factors: contains datablock(s) I. DOI: 10.1107/S1600536813004856/gw2131Isup2.hkl

Supplementary material file. DOI: 10.1107/S1600536813004856/gw2131Isup3.cml

Additional supplementary materials:  crystallographic information; 3D view; checkCIF report

## Figures and Tables

**Table 1:** Hydrogen-bond geometry (Å, °)

*D*—H⋯*A*	*D*—H	H⋯*A*	*D*⋯*A*	*D*—H⋯*A*
N1—H1⋯O2^i^	0.87 (2)	2.21 (2)	3.081 (2)	174 (2)
O2—H2⋯O4^ii^	0.82	1.76	2.572 (2)	172
C2—H2*A*⋯O1^iii^	0.93	2.63	3.536	166
C5—H5⋯O3^iv^	0.93	2.69	3.333	127

Symmetry codes: (i) 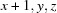; (ii) 
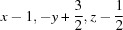
; (iii) 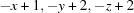; (iv) 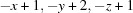.

**Fig. 1.**
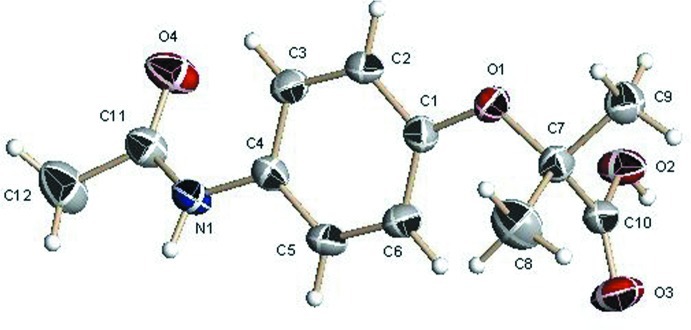
The molecular structure of (I), showing the atom-labelling scheme. Displacement ellipsoids are drawn at the 50% probability level and H atoms are shown as small spheres of arbitrary radius.

**Fig. 2.**
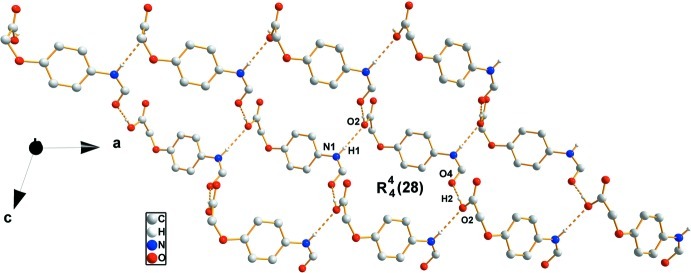
A view of the N—H···O and O—H···O interactions (dashed lines), showing the R44(28) graph set motifs. The methyl groups and hydrogen atoms not involved in hydrogen bonding have been omitted for clarity.

## supplementary crystallographic information

### Comment

Fibrates, such as bezafibrate, clofibrate and fenofibrate, which are ligands for the nuclear receptor PPAR α (Peroxisome Proliferator-Activated Receptor), are used as therapeutic agents in the treatment of dyslipidemia, heart disease and diabetic complications in humans (Forcheron *et al.*,2002). The fibrate pharmacophore has been of interest to medicinal chemists, and it is a widely used class of lipid-modifying agents that decrease plasma triglycerides (Thorp & Waring, 1962; Miller & Spence, 1998). On the other hand, paracetamol is broadly used as over-the-counter analgesic and antipyretic agent (Kis *et al.*, 2005). In order to assist our knowledge about the stereo electronic requirements from these kinds of molecules to shown antihyperlipidemic activity, we have synthesized and determined the crystal structure of a closed-related nitrofibrate analogue (Navarrete-Vázquez *et al.*, 2008), as well as the compound ethyl 2-[4-(acetylamino)phenoxy]-2-methylpropanoate, which is a bioisoster of clofibrate, with an acetamide group instead of chlorine atom (Navarrete-Vázquez *et al.*,2011). The last structure resembles to paracetamol, a well known analgesic and antipyretic agent. In this case, the hydrolysis product was obtained in order to find a new biologically active chemical entity.

In (I), all bond lengths and angles show normal values.

In the crystal structure, neighboring molecules are linked through N—H···O, O—H···O hydrogen bonds (Jeffrey, 1997) and weak C—H···O hydrogen bonds (Desiraju, 1996) forming a three dimensional network, Table 1. In the hydrogen-bond pattern, the N—H···O and O—H···O hydrogen bonds are forming corrugated sheets. These sheets are composed of *R*_4_^4^(28) graph set motifs (Bernstein, *et al.*, 1995), (Fig. 2, Table 1). Neighboring sheets are further linked by weak C—H···O hydrogen bonds, generating the three dimensional network.

### Experimental

Paracetamol (1 g, 0.0066 mol) and potassium carbonate (2 g, 0.014 mol) were dissolved in the minimum amount of dimethyl sulfoxide and were heated at 40 °C. After 20 minutes, the ethyl 2-bromo-2-methylpropionate (1.45 ml, 0.0099 mol) was added dropwise and the reaction mixture was heated to reflux (80 °C) and monitored by TLC. After the reaction completion (15 h), the reaction mixture was filtered and solid residue was washed off with acetone (10 ml). The total mother liquors were concentrated under reduced pressure and then poured into water and extracted with ethyl acetate (3 *x* 15 ml). The organic layer was dried over anhydrous Na_2_SO_4_ and partially evaporated under reduced pressure.

The resulting solid was treated with a mixture of THF/MeOH/H_2_O (3:2:1,*v*/*v*/v, 6 ml/mmol), and LiOH was added (3 equiv). The mixture stirred at room temperature for 3 h. Then, HCl solution (10% *v*/*v*) was added, and most of the organic solvents removed *in vacuo*. The partly solid residue was extracted with CH_2_Cl_2_ (3 *x* 10 ml), dried with Na_2_SO_4_, filtered, and concentrated in vacuo to give a white solid (m.p. 438 K). Single crystals were obtained from methanol. ^1^H NMR data (200 MHz; DMSO-*d*_6_; Me_4_Si) δ: 1.46 (6*H*, s, H-9 and H-10), 2.10 (3*H*, s, CH_3_CO), 6.78 (2*H*, d, *J* = 8.7, H-2 and H-6), 7.44 (2*H*, d, *J* = 8.7, H-3 and H-5), 9.83 (1*H*, bs, N—H). ^13^C NMR (50 MHz, DMSO-*d*_6_) δ: 23.8 (CH_3_CO), 25.1 (gem-di CH_3_), 78.7 (C-7), 119.5 (C-2, C-6), 120.2 (C-3, C-5), 133.9 (C-4), 161.8 (C-1), 167.9 (CONH), 175.2 (COOH). EI—MS: *m/z* (*rel*. int.) 237 (*M*^+^, 25%).

### Refinement

H atoms were positioned geometrically and constrained using the riding-model approximation [*C*—H_aryl_ = 0.93 Å, *U*_iso_(H_aryl_)= 1.2 *U*_eq_(C); *C*—H_methyl_ = 0.96 Å, *U*_iso_(H_methyl_)= 1.5 *U*_eq_(C); *O*—H_hydroxyl_ = 0.82 Å, *U*_iso_(H_hydroxyl_) = 1.5 *U*_eq_(O) = 1.5]. The hydrogen atom bonded to N1 was located by difference Fourier map. Its coordinates were refined with a distance restraint: N—H = 0.86 Å and [*U*_iso_(H) = 1.2 *U*_eq_(N)].

### Crystal data

**Table:**

C_12_H_15_NO_4_	*D*_x_ = 1.272 Mg m^−^^3^
*M**_r_* = 237.25	Melting point: 438 K
Monoclinic, *P*2_1_/*c*	Mo *K*α radiation, λ = 0.71073 Å
*a* = 8.3184 (4) Å	Cell parameters from 9500 reflections
*b* = 13.1554 (6) Å	θ = 3.0–29.3°
*c* = 12.0452 (5) Å	µ = 0.10 mm^−^^1^
β = 109.959 (5)°	*T* = 293 K
*V* = 1238.96 (10) Å^3^	Prism, colourless
*Z* = 4	0.19 × 0.14 × 0.13 mm
*F*(000) = 504	

### Data collection

**Table:**

Agilent Xcalibur Atlas Gemini diffractometer	2179 independent reflections
Radiation source: (Mo) X-ray Source	1738 reflections with *I* > 2σ(*I*)
Graphite monochromator	*R*_int_ = 0.045
Detector resolution: 10.3659 pixels mm^-1^	θ_max_ = 25.0°, θ_min_ = 3.0°
ω scans	*h* = −9→9
Absorption correction: multi-scan (*CrysAlis PRO*; Agilent, 2011)	*k* = −15→15
*T*_min_ = 0.982, *T*_max_ = 0.988	*l* = −14→14
34747 measured reflections	

### Refinement

**Table:**

Refinement on *F*^2^	Primary atom site location: structure-invariant direct methods
Least-squares matrix: full	Secondary atom site location: difference Fourier map
*R*[*F*^2^ > 2σ(*F*^2^)] = 0.037	Hydrogen site location: inferred from neighbouring sites
*wR*(*F*^2^) = 0.098	H atoms treated by a mixture of independent and constrained refinement
*S* = 1.04	*w* = 1/[σ^2^(*F*_o_^2^) + (0.0477*P*)^2^ + 0.3201*P*] where *P* = (*F*_o_^2^ + 2*F*_c_^2^)/3
2179 reflections	(Δ/σ)_max_ < 0.001
161 parameters	Δρ_max_ = 0.20 e Å^−^^3^
1 restraint	Δρ_min_ = −0.18 e Å^−^^3^

### Special details

**Table:**

Geometry. All e.s.d.'s (except the e.s.d. in the dihedral angle between two l.s. planes) are estimated using the full covariance matrix. The cell e.s.d.'s are taken into account individually in the estimation of e.s.d.'s in distances, angles and torsion angles; correlations between e.s.d.'s in cell parameters are only used when they are defined by crystal symmetry. An approximate (isotropic) treatment of cell e.s.d.'s is used for estimating e.s.d.'s involving l.s. planes.
Refinement. Refinement of *F*^2^ against ALL reflections. The weighted *R*-factor *wR* and goodness of fit *S* are based on *F*^2^, conventional *R*-factors *R* are based on *F*, with *F* set to zero for negative *F*^2^. The threshold expression of *F*^2^ > σ(*F*^2^) is used only for calculating *R*-factors(gt) *etc*. and is not relevant to the choice of reflections for refinement. *R*-factors based on *F*^2^ are statistically about twice as large as those based on *F*, and *R*- factors based on ALL data will be even larger.

### Fractional atomic coordinates and isotropic or equivalent isotropic displacement parameters (Å^2^)

**Table:**

	*x*	*y*	*z*	*U*_iso_*/*U*_eq_	
C1	0.57142 (18)	0.98467 (12)	0.81385 (13)	0.0332 (3)	
C2	0.67842 (19)	0.96019 (12)	0.92728 (13)	0.0350 (4)	
H2A	0.6433	0.9739	0.9912	0.042*	
C3	0.83553 (19)	0.91600 (12)	0.94636 (13)	0.0355 (4)	
H3	0.9059	0.9006	1.0229	0.043*	
C4	0.88934 (18)	0.89431 (11)	0.85144 (13)	0.0320 (3)	
C5	0.7820 (2)	0.91711 (13)	0.73829 (13)	0.0397 (4)	
H5	0.8165	0.9023	0.6744	0.048*	
C6	0.6234 (2)	0.96183 (13)	0.71873 (13)	0.0419 (4)	
H6	0.5523	0.9764	0.6422	0.050*	
C7	0.29399 (19)	1.06405 (12)	0.69952 (13)	0.0347 (4)	
C8	0.3597 (3)	1.15075 (14)	0.64257 (18)	0.0578 (5)	
H8A	0.4046	1.2038	0.6995	0.087*	
H8B	0.2675	1.1771	0.5768	0.087*	
H8C	0.4484	1.1259	0.6155	0.087*	
C9	0.1450 (2)	1.10112 (13)	0.73554 (14)	0.0421 (4)	
H9A	0.1112	1.0482	0.7779	0.063*	
H9B	0.0504	1.1185	0.6662	0.063*	
H9C	0.1798	1.1599	0.7852	0.063*	
C10	0.23247 (19)	0.97382 (12)	0.61524 (12)	0.0349 (4)	
C11	1.1489 (2)	0.79383 (12)	0.95558 (14)	0.0400 (4)	
C12	1.3223 (2)	0.76481 (17)	0.95380 (19)	0.0633 (6)	
H12A	1.4078	0.7842	1.0271	0.095*	
H12B	1.3434	0.7990	0.8897	0.095*	
H12C	1.3267	0.6926	0.9434	0.095*	
H1	1.099 (3)	0.8691 (17)	0.8137 (17)	0.076*	
N1	1.05465 (16)	0.85358 (10)	0.86741 (11)	0.0362 (3)	
O1	0.41935 (13)	1.03044 (9)	0.80935 (8)	0.0390 (3)	
O2	0.20332 (16)	0.89154 (9)	0.66919 (9)	0.0454 (3)	
H2	0.1674	0.8458	0.6208	0.068*	
O3	0.20982 (17)	0.97740 (11)	0.51131 (9)	0.0566 (4)	
O4	1.09585 (17)	0.76373 (10)	1.03443 (11)	0.0555 (4)	

### Atomic displacement parameters (Å^2^)

**Table:**

	*U*^11^	*U*^22^	*U*^33^	*U*^12^	*U*^13^	*U*^23^
C1	0.0302 (8)	0.0358 (8)	0.0335 (8)	−0.0015 (6)	0.0109 (6)	−0.0033 (6)
C2	0.0367 (8)	0.0422 (9)	0.0286 (7)	0.0008 (7)	0.0145 (6)	−0.0035 (7)
C3	0.0374 (8)	0.0405 (9)	0.0279 (7)	0.0021 (7)	0.0101 (6)	−0.0001 (7)
C4	0.0341 (8)	0.0301 (8)	0.0351 (8)	−0.0017 (6)	0.0161 (6)	0.0002 (6)
C5	0.0417 (9)	0.0518 (10)	0.0307 (8)	0.0021 (8)	0.0190 (7)	0.0000 (7)
C6	0.0401 (9)	0.0567 (11)	0.0282 (8)	0.0039 (8)	0.0106 (7)	0.0009 (7)
C7	0.0328 (8)	0.0373 (9)	0.0325 (8)	0.0009 (7)	0.0092 (6)	0.0047 (7)
C8	0.0660 (13)	0.0443 (11)	0.0709 (13)	−0.0051 (9)	0.0333 (10)	0.0093 (9)
C9	0.0390 (9)	0.0435 (9)	0.0423 (9)	0.0073 (7)	0.0121 (7)	0.0020 (7)
C10	0.0314 (8)	0.0453 (9)	0.0281 (8)	0.0011 (7)	0.0105 (6)	0.0036 (7)
C11	0.0423 (9)	0.0361 (9)	0.0462 (9)	0.0052 (7)	0.0210 (8)	0.0040 (7)
C12	0.0519 (11)	0.0667 (13)	0.0807 (14)	0.0208 (10)	0.0346 (11)	0.0217 (11)
N1	0.0377 (7)	0.0386 (7)	0.0385 (7)	0.0047 (6)	0.0211 (6)	0.0056 (6)
O1	0.0312 (6)	0.0541 (7)	0.0295 (5)	0.0068 (5)	0.0073 (4)	−0.0041 (5)
O2	0.0633 (8)	0.0415 (7)	0.0362 (6)	−0.0136 (6)	0.0231 (6)	−0.0078 (5)
O3	0.0713 (9)	0.0697 (9)	0.0273 (6)	−0.0061 (7)	0.0149 (6)	0.0010 (6)
O4	0.0630 (8)	0.0584 (8)	0.0571 (7)	0.0249 (6)	0.0362 (7)	0.0247 (6)

### Geometric parameters (Å, º)

**Table:**

C1—O1	1.3852 (18)	C8—H8A	0.9600
C1—C6	1.389 (2)	C8—H8B	0.9600
C1—C2	1.390 (2)	C8—H8C	0.9600
C2—C3	1.376 (2)	C9—H9A	0.9600
C2—H2A	0.9300	C9—H9B	0.9600
C3—C4	1.392 (2)	C9—H9C	0.9600
C3—H3	0.9300	C10—O3	1.2015 (17)
C4—C5	1.383 (2)	C10—O2	1.3268 (19)
C4—N1	1.4264 (19)	C11—O4	1.2410 (18)
C5—C6	1.389 (2)	C11—N1	1.339 (2)
C5—H5	0.9300	C11—C12	1.499 (2)
C6—H6	0.9300	C12—H12A	0.9600
C7—O1	1.4461 (18)	C12—H12B	0.9600
C7—C8	1.525 (2)	C12—H12C	0.9600
C7—C9	1.526 (2)	N1—H1	0.86 (2)
C7—C10	1.532 (2)	O2—H2	0.8200
			
O1—C1—C6	126.82 (13)	C7—C8—H8C	109.5
O1—C1—C2	114.14 (12)	H8A—C8—H8C	109.5
C6—C1—C2	119.04 (14)	H8B—C8—H8C	109.5
C3—C2—C1	120.99 (13)	C7—C9—H9A	109.5
C3—C2—H2A	119.5	C7—C9—H9B	109.5
C1—C2—H2A	119.5	H9A—C9—H9B	109.5
C2—C3—C4	120.18 (14)	C7—C9—H9C	109.5
C2—C3—H3	119.9	H9A—C9—H9C	109.5
C4—C3—H3	119.9	H9B—C9—H9C	109.5
C5—C4—C3	118.96 (14)	O3—C10—O2	123.53 (15)
C5—C4—N1	118.86 (13)	O3—C10—C7	123.93 (14)
C3—C4—N1	122.12 (13)	O2—C10—C7	112.52 (12)
C4—C5—C6	121.01 (14)	O4—C11—N1	121.96 (14)
C4—C5—H5	119.5	O4—C11—C12	121.67 (15)
C6—C5—H5	119.5	N1—C11—C12	116.36 (14)
C1—C6—C5	119.80 (14)	C11—C12—H12A	109.5
C1—C6—H6	120.1	C11—C12—H12B	109.5
C5—C6—H6	120.1	H12A—C12—H12B	109.5
O1—C7—C8	112.51 (14)	C11—C12—H12C	109.5
O1—C7—C9	103.87 (11)	H12A—C12—H12C	109.5
C8—C7—C9	109.84 (14)	H12B—C12—H12C	109.5
O1—C7—C10	110.04 (12)	C11—N1—C4	127.18 (12)
C8—C7—C10	111.79 (13)	C11—N1—H1	116.3 (15)
C9—C7—C10	108.42 (12)	C4—N1—H1	116.5 (15)
C7—C8—H8A	109.5	C1—O1—C7	122.17 (11)
C7—C8—H8B	109.5	C10—O2—H2	109.5
H8A—C8—H8B	109.5		
			
O1—C1—C2—C3	−178.80 (14)	O1—C7—C10—O2	43.10 (16)
C6—C1—C2—C3	1.4 (2)	C8—C7—C10—O2	168.89 (14)
C1—C2—C3—C4	−0.5 (2)	C9—C7—C10—O2	−69.88 (16)
C2—C3—C4—C5	−0.5 (2)	O4—C11—N1—C4	2.6 (3)
C2—C3—C4—N1	176.59 (14)	C12—C11—N1—C4	−177.66 (16)
C3—C4—C5—C6	0.6 (2)	C5—C4—N1—C11	−153.32 (16)
N1—C4—C5—C6	−176.60 (15)	C3—C4—N1—C11	29.6 (2)
O1—C1—C6—C5	178.93 (15)	C6—C1—O1—C7	−2.1 (2)
C2—C1—C6—C5	−1.3 (2)	C2—C1—O1—C7	178.16 (13)
C4—C5—C6—C1	0.3 (3)	C8—C7—O1—C1	−65.53 (19)
O1—C7—C10—O3	−138.24 (15)	C9—C7—O1—C1	175.74 (13)
C8—C7—C10—O3	−12.4 (2)	C10—C7—O1—C1	59.85 (17)
C9—C7—C10—O3	108.79 (17)		

### Hydrogen-bond geometry (Å, º)

**Table:**

*D*—H···*A*	*D*—H	H···*A*	*D*···*A*	*D*—H···*A*
N1—H1···O2^i^	0.87 (2)	2.21 (2)	3.081 (2)	174 (2)
O2—H2···O4^ii^	0.82	1.76	2.572 (2)	172
C3—H3···O4	0.93	2.37	2.874 (2)	114
C2—H2*A*···O1^iii^	0.93	2.63	3.536	166
C5—H5···O3^iv^	0.93	2.69	3.333	127

Symmetry codes: (i) *x*+1, *y*, *z*; (ii) *x*−1, −*y*+3/2, *z*−1/2; (iii) −*x*+1, −*y*+2, −*z*+2; (iv) −*x*+1, −*y*+2, −*z*+1.
